# Clinical characteristics of a large cohort of patients with narcolepsy candidate for pitolisant: a cross-sectional study from the Italian PASS Wakix® Cohort

**DOI:** 10.1007/s10072-022-06210-9

**Published:** 2022-06-25

**Authors:** Carlotta Mutti, Valerio Brunetti, Michela Figorilli, Claudio Liguori, Fabio Pizza, Paola Proserpio, Tommaso Sacco, Giuseppe Pedrazzi, Isabelle Lecomte, Nora Blanchard, Elio Clemente Agostoni, Enrica Bonanni, Diego Centonze, Alessandro Cicolin, Giacomo Della Marca, Luigi Ferini-Strambi, Raffaele Ferri, Gian Luigi Gigli, Francesca Izzi, Rocco Liguori, Raffaele Lodi, Lino Nobili, Liborio Parrino, Fabio Placidi, Monica Puligheddu, Andrea Romigi, Maria Antonietta Savarese, Michele Terzaghi, Giuseppe Plazzi

**Affiliations:** 1 Sleep Disorders Center, Department of General and Specialist Medicine, Parma University Hospital, Parma, Italy; 2 Sleep Medicine Unit, UOC di Neurologia, Dipartimento Scienze dell’invecchiamento, neurologiche, ortopediche e della testa-collo, Fondazione Policlinico Universitario A. Gemelli IRCCS, Largo A. Gemelli 8, 00168 Rome, Italy; 3 Department of Neurosciences, Università Cattolica del Sacro Cuore, Largo Francesco Vito 1, 00168 Rome, Italy; 4 Sleep Disorder Research Center, Department of Medical Sciences and Public Health, University of Cagliari, Cagliari, Italy; 5 Sleep Medicine Center, Neurology Unit, University Hospital of Rome Tor Vergata, Rome, Italy; 6 Department of Systems Medicine, University of Rome Tor Vergata, Rome, Italy; 7 Dipartimento di Scienze Biomediche e Neuromotorie (DIBINEM), Università di Bologna, Bologna, Italy; 8 IRCCS Istituto delle Scienze Neurologiche di Bologna, Bologna, Italy; 9 Sleep Medicine Center, Neurology and Stroke Unit, Department of Neuroscience, ASST Grande Ospedale Metropolitano Niguarda, Milan, Italy; 10 Bioprojet Italia, Medical Department, Via Giovan Battista Pirelli 11, Milan, Italy; 11 Department of Medicine and Surgery, Unit of Neuroscience, Interdepartmental Center of Robust Statistics (Ro.S.A.), University of Parma, Parma, Italy; 12 Bioprojet, Study Medical Director, 9 Rue Rameau, Paris, France; 13 Bioprojet, Pharmacovigilance Department, 9 Rue Rameau, Paris, France; 14 Sleep Disorder Center, University of Pisa, Pisa, Italy; 15 Unit of Neurology, IRCCS Neuromed, Pozzilli, IS Italy; 16 Centro di Medicina del Sonno, Dipartimento di Neuroscienze Rita Levi Montalcini, Università di Torino, AOU Città della Salute e della Scienza, Turin, Italy; 17 Università Vita-Salute San Raffaele, Milan, Italy; 18 Sleep Research Center, Department of Neurology I.C, Oasi Research Institute - IRCCS, Troina, EN Italy; 19 Clinical Neurology Unit, Department of Medicine (DAME), University of Udine and Santa Maria della Misericordia University Hospital, Udine, Italy; 20 Department of Neuroscience (DINOGMI), University of Genoa, Genoa, Italy; 21 Child Neuropsychiatry Unit, Istituto G. Gaslini, Genoa, Italy; 22 Sleep Medicine Center IRCCS Neuromed, Pozzilli, IS Italy; 23 Neurology Unit and Stroke Center, Department of Basic Medical Sciences, Neurosciences and Sense Organs, University of Bari “Aldo Moro”, Bari, Italy; 24 Unit of Sleep Medicine and Epilepsy, IRCCS Mondino Foundation, Pavia, Italy; 25 Department of Brain and Behavioral Sciences, University of Pavia, Pavia, Italy; 26 Dipartimento di Scienze Biomediche, Metaboliche e Neuroscienze, Università di Modena e Reggio-Emilia, Modena, Italy; 27 Sleep Disorders, Narcolepsy and CNS Hypersomnias Center - IRCCS Istituto delle Scienze Neurologiche di Bologna, Bologna, Italy

**Keywords:** Polytherapy, Combined therapy, Pitolisant, Treatment, Sleepiness, Sleep

## Abstract

**Introduction:**

Narcolepsy is a chronic and rare hypersomnia of central origin characterized by excessive daytime sleepiness and a complex array of symptoms as well as by several medical comorbidities. With growing pharmacological options, polytherapy may increase the possibility of a patient-centered management of narcolepsy symptoms. The aims of our study are to describe a large cohort of Italian patients with narcolepsy who were candidates for pitolisant treatment and to compare patients’ subgroups based on current drug prescription (drug-naïve patients in whom pitolisant was the first-choice treatment, switching to pitolisant from other monotherapy treatments, and adding on in polytherapy).

**Methods:**

We conducted a cross-sectional survey based on Italian data from the inclusion visits of the Post Authorization Safety Study of pitolisant, a 5-year observational, multicenter, international study.

**Results:**

One hundred ninety-one patients were enrolled (76.4% with narcolepsy type 1 and 23.6% with narcolepsy type 2). Most patients (63.4%) presented at least one comorbidity, mainly cardiovascular and psychiatric. Pitolisant was prescribed as an add-on treatment in 120/191 patients (62.8%), as switch from other therapies in 42/191 (22.0%), and as a first-line treatment in 29/191 (15.2%). Drug-naive patients presented more severe sleepiness, lower functional status, and a higher incidence of depressive symptoms.

**Conclusion:**

Our study presents the picture of a large cohort of Italian patients with narcolepsy who were prescribed with pitolisant, suggesting that polytherapy is highly frequent to tailor a patient-centered approach.

## Introduction

Narcolepsy is a life-long disabling neurological disorder characterized by excessive daytime sleepiness (EDS) with REM sleep occurring at sleep onset (SOREMP) and other dissociated REM-sleep/wake symptoms including cataplexy and nocturnal sleep disruption. Narcolepsy includes two different disease entities, narcolepsy type 1 (NT1) and type 2 (NT2). NT1 is a well-defined disease entity due to loss of orexin-producing neurons of the lateral hypothalamus, most probably of autoimmune origin, and it is associated with cataplexy and/or low or absent cerebrospinal fluid (CSF) orexin levels [1]. Conversely, NT2 is a less defined disease entity sharing with NT1 the high sleep propensity with SOREMPs, but in the absence of cataplexy and with evidence (if lumbar puncture is performed) of normal CSF orexin levels.

Comorbid psychiatric disorders, such as mood disturbances, attention-deficit/hyperactivity disorder, eating disorders, and anxiety, increase the burden of narcolepsy patients and contribute to a more severe disease burden [2]. Narcolepsy patients suffer also from severe cardiovascular comorbidity, and it is unknown to which extent the disease per se and the long-term pharmacological treatments increase the risk of acute vascular events, calling for a regular assessment of cardiovascular health [3].

As for numerous rare diseases, underdiagnosis or late and even misdiagnosis are common, with diagnostic delay reaching up to 14 years [4, 5], with negative consequences on patients’ wellbeing and health during diagnostic delay: lower quality of life, psychological distress, higher unemployment, and increased road accident risk [6].

Although NT1 symptoms arise primarily from a loss of orexin neurons in the lateral hypothalamus, the involvement of neural circuits, including histaminergic, noradrenergic, dopaminergic, and serotoninergic pathways, is also well established given the widespread interactions of the hypocretinergic system within the central nervous system [7].

The European Medicine Agency (EMA) authorized pharmacological approaches such as modafinil and solriamfetol only for EDS in narcolepsy. Pitolisant is indicated for both NT1 and NT2, and sodium oxybate only for NT1, given their efficacy on cataplexy. Tricyclic antidepressants, selective serotonin reuptake inhibitors (SSRI), and serotonin-norepinephrine reuptake inhibitors (SNRI) are widely used as off-label therapies to improve cataplexy despite the absence of evidence-based studies [8–10]. A specific methylphenidate brand is indicated for narcolepsy in some European countries, but not in Italy. The availability of several drugs with distinct effects requires a careful weighing of the efficacy/safety balance of each approach and, at the same time, paves the way for tailored combination therapies. Several studies [11–20] addressed the use of drug combination in the management of narcolepsy in both adults and children. Recent guidelines for narcolepsy reported the need for polytherapy on the basis of symptom combinations and response across disease management [21], a due update consistent with the real-world disease management.

Post Authorization Safety Study (PASS)-pitolisant, requested by the EMA’s Committee for Medicinal Products for Human Use (CHMP), is a 5-year observational, multicenter, international study, started in 2017, and aimed at documenting the long-term safety and management of the drug in routine medical practice. The main aim of this study is to present the clinical picture of a large cohort of Italian narcolepsy patients who were candidates for pitolisant treatment (enrolled in the PASS-pitolisant by tertiary Sleep Medicine Centers) and to compare patients’ subgroups based on current drug prescription (drug-naïve patients in whom pitolisant was the first-choice treatment, switching to pitolisant from other monotherapy treatments, and adding on in polytherapy).

## Materials and methods

### Study design and setting

**Fig. 1:**
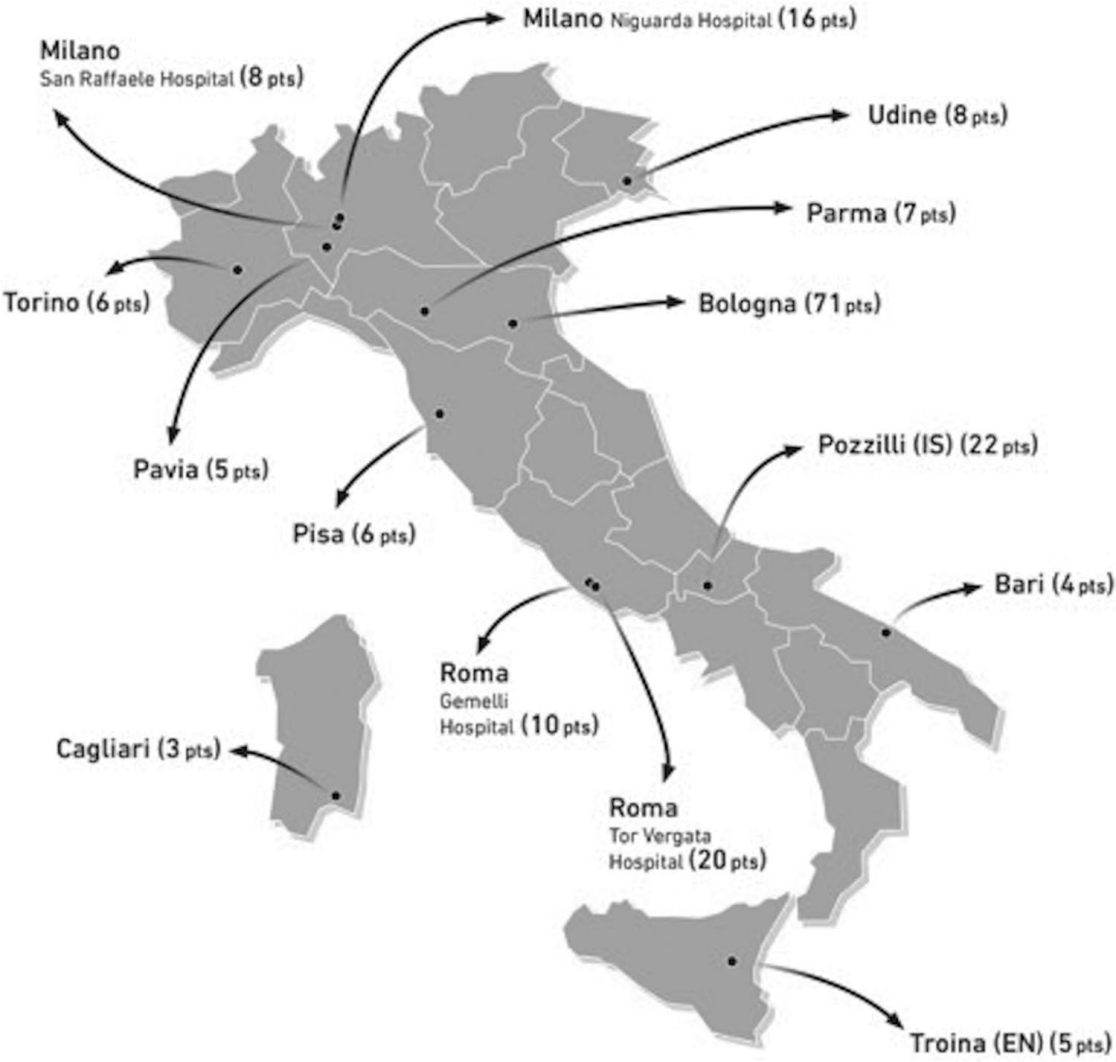
Sleep centers participating in the study with the number of patients enrolled for each center

The PASS-pitolisant study is an international, multicenter, observational, prospective, open-label long-term post-authorization safety study, currently ongoing in sleep centers with expertise in the management of narcolepsy. The enrollment period started in December 2016, in Italy in April 2017, and ended in June 2019, except for the UK, where the enrollment period was extended until 31 October 2019. In this paper, we present a cross-sectional study based on data from the inclusion visits performed in 14 Italian sleep centers that were included in the third interim report of the study updated as of January 2, 2020; therefore, none of patients was under pitolisant at the time of data analysis. Sleep centers participating in the study with the number of patients enrolled for each center are reported in Fig. 1.

The decision to initiate treatment with pitolisant was taken by the physician, according to routine clinical practice for a patient/symptom-centered approach, and independently of the decision to enroll the patient into the study. The research protocol was approved in each site by the local ethics committee.

### Participants and data sources

Eligible patients were aged ≥ 18 years with NT1 or NT2 narcolepsy and suitable to be prescribed with pitolisant by specialists, according to clinical judgment and following the prescribing information of the drug’s summary of product characteristics, consistent with the real-world disease management. The exclusion criteria were (1) known contraindication to pitolisant (hypersensitivity to the active substance or to any of the excipients, severe hepatic insufficiency, breastfeeding, and pregnancy) and (2) patients who had received pitolisant during the 12 months preceding the inclusion. Before enrollment, patients signed informed consent to participate in the study. Study visits and/or questionnaire-based re-assessments were scheduled after patients’ inclusion, according to each sleep center’s routine practice, with the minimum requirement of one yearly visit over the course of treatment (in line with the clinical need to confirm drug prescription in Italy). Data collection was performed both with an e-CRF completed by the investigators and a self-administered paper questionnaire answered by patients at each visit. Data were collected locally, while data analysis was performed centrally by a dedicated contract research organization. In the current research, only data from the baseline visit were analyzed. The study protocol provided for annual meetings during which the investigators shared the patients’ treatment strategies.

The following data were recorded for each patient: demographics (age and sex), vital signs (heart rate, blood pressure, weight, height, and BMI), and comorbidities including cardiovascular diseases (coronary or cardiac insufficiency, arrhythmia, hypertension, and other cardiac/vascular disorders), hypercolesterolemia, neurological and psychiatric diseases (depression, anxiety, and other neurological or psychiatric conditions), obstructive sleep apnea, renal impairment, hepatic impairment, diabetes, obesity, autoimmune diseases, and other types of comorbidities. For each patient, we collected the diagnosis (NT1 or NT2), age at diagnosis, disease duration, diagnostic methodology (polysomnography plus multiple sleep latency test; CSF orexin deficiency), and the presence of symptoms (i.e., cataplexy, sleep paralysis, hallucinations, automatic behaviors, and disrupted nocturnal sleep). Daytime sleepiness at study inclusion was evaluated by the Epworth Sleepiness Scale (ESS) [22], and a score > 10 is consistent with the presence/persistence of EDS. To investigate the prevalence and impact of depressive symptoms, each patient completed the short version of the Beck Depression Inventory (BDI-13) [23], a self-administered form consisting of 13 items, scored from 0 to 3, and evaluating the main components of depression (affective, somatic, and cognitive), with a score > 4 suggestive of the presence of depressive symptoms (5–7: mild depressive symptoms; 8–15: moderate depressive symptoms; ≥16 severe depressive symptoms). Quality of life was investigated by EQ-5D-5L [24], a brief health status measure with five questions with Likert response options and a visual analog scale (EQ-VAS) where the patients are asked to rate their own health from 0 to 100 (the worst and best imaginable health, respectively). The descriptive part of the test evaluated 5 dimensions of health (mobility, self-care, usual activities, pain or discomfort, and anxiety or depression), ranking them into five levels of severity (no problems, slight problems, moderate problems, severe problems, and unable to perform or extreme problems). The burden of sleepiness-related impairment on the ability to conduct daily and recreational activities was evaluated by means of the short version of the Functional Outcomes of Sleep Questionnaire (FOSQ-10) [25], a brief and validated self-administered questionnaire consisting of 10 items with a 4-point Likert response format, which investigates the impact of sleepiness on five main domains (general productivity, activity level, vigilance, social outcomes, and intimate and sexual relationships), with higher scores representing better status. The severity of EDS and cataplexy were also evaluated by means of the Clinical Global Impression severity scale (CGI-S) [ 26], a 7-point scale (ranging from “normal” to “among the most extremely ill patients”) that requires the clinician to score the severity of the patient’s illness at the time of assessment.

Concomitant medications were recorded for each patient, with particular regard for specific treatment of EDS (modafinil, sodium oxybate, and methylphenidate) and cataplexy (sodium oxybate, antidepressants). None of the patients included in the current study was under treatment with solriamfetol as long, as at the time of study enrollment, the drug was not yet available (EMA’s authorization on January 2020).

### Statistical analysis

Continuous variables are presented as means and standard deviation and as median and interquartile range, as appropriate; categorical variables are presented as absolute frequencies and percentages. Statistical analysis was performed in multiple steps. First, the study cohort was divided into three subgroups, according to the modality of prescription of pitolisant: patients who still were not under anti-narcoleptic medication at the inclusion visit and in whom pitolisant was prescribed as the first-choice treatment (“naive group”), patients that stopped their prior narcoleptic medication while starting pitolisant therapy after the inclusion visit (“switched group”), and patients that continued at least one prior treatment together with pitolisant (“add-on group”). A univariate comparison for categorical variables was performed on these data using the *χ*^2^ test; Fisher’s exact test was used for post hoc analysis. Continuous variables were compared by one-way ANOVA, and post hoc comparisons were carried out by means of Tukey’s test. Appropriate multiple comparison corrections were applied to take family-wise type-I errors into account.

Subsequently, to adjust for the effects of potential confounders, multivariate multinominal logistic regression was used to model the probabilities of the different modalities of prescription of pitolisant. Backward selection was used to identify significant clinical baseline dependent variables, among clinically relevant variables with *p* ≤ 0.25 with univariate analysis (NT1, ESS>10, BDI, VAS, QoL, FOSQ-10), adjusted for age and sex. The level of significance was set at *p* < 0.05. Statistical analyses were carried out with SAS software (version 9.4, SAS Institute, North Carolina, USA).

## Results

### Demographic and clinical data

A total of 191 narcoleptic patients (52.4% males; mean age: 42.5±16.1 years) were enrolled by the 14 Italian sleep centers participating in the PASS-pitolisant study (see Fig. 1).

Most of the patients (146/191, 76.4%) were affected by NT1. The mean age at narcolepsy diagnosis was 35.5±15.8 years (range 7.8–80.4) and the mean disease duration was 7.1±6.7 years (range 0–31.9).

EDS (ESS > 10) was the commonest reported symptom (164/191, 85.9%; median ESS score: 15; IQR: 12–19; range: 4–24). EDS was classified as severe (ESS > 15) in almost half of the patients (91/191, 47.6%). Cataplexy was reported in 74.3% of cases. Notably, a relatively small number of patients (27/191, 14.1%) reported an ESS score of ≤ 10. In these cases, pitolisant was prescribed in order to improve cataplexy.

Results of nocturnal polysomnography plus multiple sleep latency test were available for 187/191 (97.9%) patients, and in 167/191 (87.4%), the latter showed a mean sleep latency ≤ 8 min and at least two SOREMPs. In the remaining cases, the diagnosis was established according to the presence of EDS and CSF orexin-A concentration below the cut-off value of 110 pg/mL.

Orexin-A levels were measured in 97 out of 191 (50.8%) patients, and 59 out of 97 (60.8%) had CSF orexin-A concentration below the cut-off value of 110 pg/mL, consistent with a diagnosis of NT1, including a patient without clinical history of cataplexy.

The commonest associated narcoleptic symptoms were disrupted nocturnal sleep (69.1%), sleep hallucinations (64.4%), sleep paralysis (60.2%), and automatic behaviors (37.7%).

### Patients’ self-assessment of depression, quality of life, and disease burden

According to the BDI scores, 52.2% presented depressive symptoms (16.0%, 27.7%, and 8.5% of mild, moderate, and severe intensity, respectively).

Results of EQ-5D-5L showed that most patients did not complain of problems in mobility, self-care, or pain/discomfort dimensions; conversely, around half of the patients’ reported problems in pursuing their usual activities and/or reported anxiety/depressive mood. The mean EQ-VAS value was 64.7 ± 20.7 (range 10.0–100.0); the mean FOSQ-10 score was 13.7 ± 3.8 (range 5.0–20.0).

The main demographic and clinical characteristics are summarized in Table 1.

**Table 1:** Clinical and demographic characteristics of the study group

	n**°**	%	Mean	SD	Median	IQR
Age (years)			42.5	16.1	42.7	27.9–54.5
Sex (male, n°)	100	52.4				
BMI (kg/m^2^)			27.3	5.5	26.5	23.4–30.9
NT1 (n°)	146	76.4				
Age at diagnosis (years)			35.5	15.8	33.3	22.5–48.2
Mean disease duration (years)			7.1	6.7	6	0.8–11.3
EDS (ESS > 10) (n°)	164	85.9				
ESS (score)			15.2	4.4	15	12–19
Cataplexy (n°)	142	74.3				
Hypocretin deficiency (n°)*	59	60.8				
Sleep paralysis (n°)	115	60.2				
Sleep hallucinations (n°)	123	64.4				
Automatic behaviors (n°)	72	37.7				
Disrupted nocturnal sleep (n°)	132	69.1				
BDI (score)			6.4	6.0	5.0	2.0–10.0
Mild depressive symptoms (BDI 5–7) (n°)	30	16.0				
Moderate depressive symptoms (BDI 8–15) (n°)	52	27.7				
Severe depressive symptoms (BDI 15-39) n°	16	8.5				
EQ-5D-5L (in health) (n°)	54	29.2				
EQ-VAS Health (score)			64.7	20.7	70.0	50.0–80.0
FOSQ10 (score)			13.7	3.8	14.5	10.9–16.7

*BDI*, short version of the Beck Depression Inventory; *BMI*, body mass index; *EDS*,

excessive daytime sleepiness; *EQ-5D-5L*, 5-level EQ-5D version; *FOSQ-10*, Functional Outcomes

of Sleep Questionnaire; *IQR*, interquartile range; *NT1*, narcolepsy type 1; *SD*, standard deviation; *VAS*, EQ visual analogue scale

^*^Data are available for 107 patients

### Disease severity assessment

Regarding EDS, the CGI-S showed that most included subjects were described as moderately ill (29.8%), markedly ill (34.6%), severely ill (15.7%), or among the most extremely ill patients (0.5%). The remaining 19.4% of patients were deemed by the sleep specialists as normal, borderline ill, or mildly ill. With regard the cataplexy CGI-S, patients were categorized as borderline ill or mildly ill in around half of the cases (46.6%), with 24.7% being moderately ill, 15.1% markedly ill, 8.2% severely ill, and 0.7% of patients described as among the most extremely ill.

### Comorbidities

Most of the patients (121/191, 63.4%) presented at least one comorbidity. The commonest comorbidity was obesity, affecting up to one-third of patients (62/191, 32.5%), followed by cardiovascular diseases (28.8%) and neurological-psychiatric disorders (24.1%). Among cardiovascular comorbidities, hypertension (16.2%) and arrhythmias (5.2%) were the most frequent. Details regarding other comorbidities are provided in Table 2.

**Table 2:** Types and prevalence of comorbidities in the study group

	n°	%
Comorbidities	121	63.4
*Cardiovascular*	55	28.8
*Depression*	17	8.9
*Anxiety*	11	5.8
*Epilepsy*	10	5.2
*Sleep apnea*	34	17.8
*Diabetes*	14	7.3
*Obesity*	62	32.5
*Autoimmune disease*	12	6.3
*Others*	30	15.7

### Previous narcolepsy treatments

Up to 84.8% of patients had received at least one prior treatment for narcolepsy. Treatment used for EDS included modafinil (79.6%), sodium oxybate (29.3%), and metylphenidate (1.0%). Medications to treat concomitant insomnia disorder were used in a minority of cases (1.6%) and included melatonin, lormetazepam, promethazine, zolpidem, and zopiclone. Medications adopted to treat cataplexy included sodium oxybate (29.3%), venlafaxine (29.3%), non-SSRIs (e.g., clomipramine, trimipramine) (2.6%), NRIs (1.6%), SSRI (1.0%), or other antidepressant therapies (3.1%). Detailed pharmacological treatment of the study cohort is reported in Table 3.

**Table 3:** Current treatments administered at the time of enrollment in the study group

	n**°**	**%**
Treatment for EDS (total)	162	84.8
*Modafinil*	152	79.6
*Sodium oxybate*	56	29.3
*Methyphanidate*	2	1.0
Treatment for cataplexy (total)	60	31.4
*Sodium oxybate*	56	29.3
*Venlafaxine*	56	29.3
*SSRIs*	2	1.0
*Non-SSRIs*	5	2.6
*NRI*	3	1.6
*Other antidepressants*	6	3.1
Number of prior treatments		
*0*	29	15.2
*1*	75	39.3
*2*	57	29.8
*≥3*	30	15.7

*EDS*, excessive daytime sleepiness; *NRI*, norepinephrine reuptake inhibitor; *SSRI*, selective serotonin reuptake inhibitor

### Treatment group comparison

Pitolisant was most commonly prescribed as an add-on treatment (120/191, 62.8%; “add-on group”), rather than switched from other therapies (42/191, 22.0%, “switched group”) or as first treatment (29/191, 15.2%, “naïve group”).

The three groups presented a significant difference in the prevalence of NT1 (*χ*^2^= 9.125, *p* = 0.010) which, in the post hoc comparison, was significantly higher in the “add-on group” (99/120, 82.5%) compared to the “switched group” (25/42, 59.5%) (*p* = 0.005, Fisher’s exact test). Similarly, the prevalence of cataplexy (*χ*^2^ = 9.222, *p* = 0.010) and of sleep paralysis (*χ*^2^ = 8.507, *p* = 0.014) differs among groups; in particular, cataplexy was significantly more frequent in the “add-on group” (97/120, 80.8%) compared to the “switched group” (24/42, 57.1%) (*p* = 0.004, Fisher’s exact test), while sleep paralysis were more commonly observed in the “naive group” (22/29, 75.9%) compared to the “switched group” (18/42, 42.9%) (*p* = 0.008, Fisher’s exact test). Also, the distribution of cases with the presence/persistence of EDS (ESS > 10) showed significant differences among the three groups (*χ*^2^ = 9.866, *p* = 0.007), being present in all patients of the “naive group” (29/29, 100%). The burden of sleepiness-related impairment on the ability to conduct daily and recreational activities, evaluated by means of FOSQ-10, significantly differs among groups (*F*_2, 188_ = 72.770, *p* = 0.001), with post hoc comparison in particular showing significantly lower values of FOSQ10, consistent with a lower status in the “naive group” compared to the “add-on group” (FOSQ10 in the “naive group”: 11.6 ± 4.0; FOSQ10 in the “add-on group”: 14.4 ± 3.4; *p*=0.001). The BDI scores also showed a difference among groups (*F*_2, 188_=32.814, *p*=0.040) as a consequence of significantly higher values in the “naive group” than in the “add-on group” (“naive group”: 9.0±7.9, “add-on group”: 6.0±5.7; *p*=0.040) and a trend towards significance in the comparison between the “naive group” and the “switched group” (“naive group”: 9.0±7.9, “switched group”: 5.8±4.9; *p*=0.068). In particular, the prevalence of “severe depressive symptoms,” defined as a BDI score greater than 15, differs among the three groups (χ^2^= 6.900, *p*=0.032) as a result of a significantly higher prevalence in the “drug-naïve group” (6/29, 21.4%) compared to “add-on group” (8/120, 6.8%) (*p*=0.031, Fisher’s exact test). Finally, the three groups differed in time from diagnosis to inclusion (*F*_2, 188_ = 142.424, *p* < 0.001), which was significantly shorter in the “naive group” compared to the “add-on group” (“naive group”: 1.6±3.6 years, “add-on group”: 8.5±6.7 years; *p* < 0.001) and to the “switched group” (“naive group”: 1.6±3.6 years, “switched group”: 6.7±6.4 years; *p* = 0.003).

**Fig. 2:**
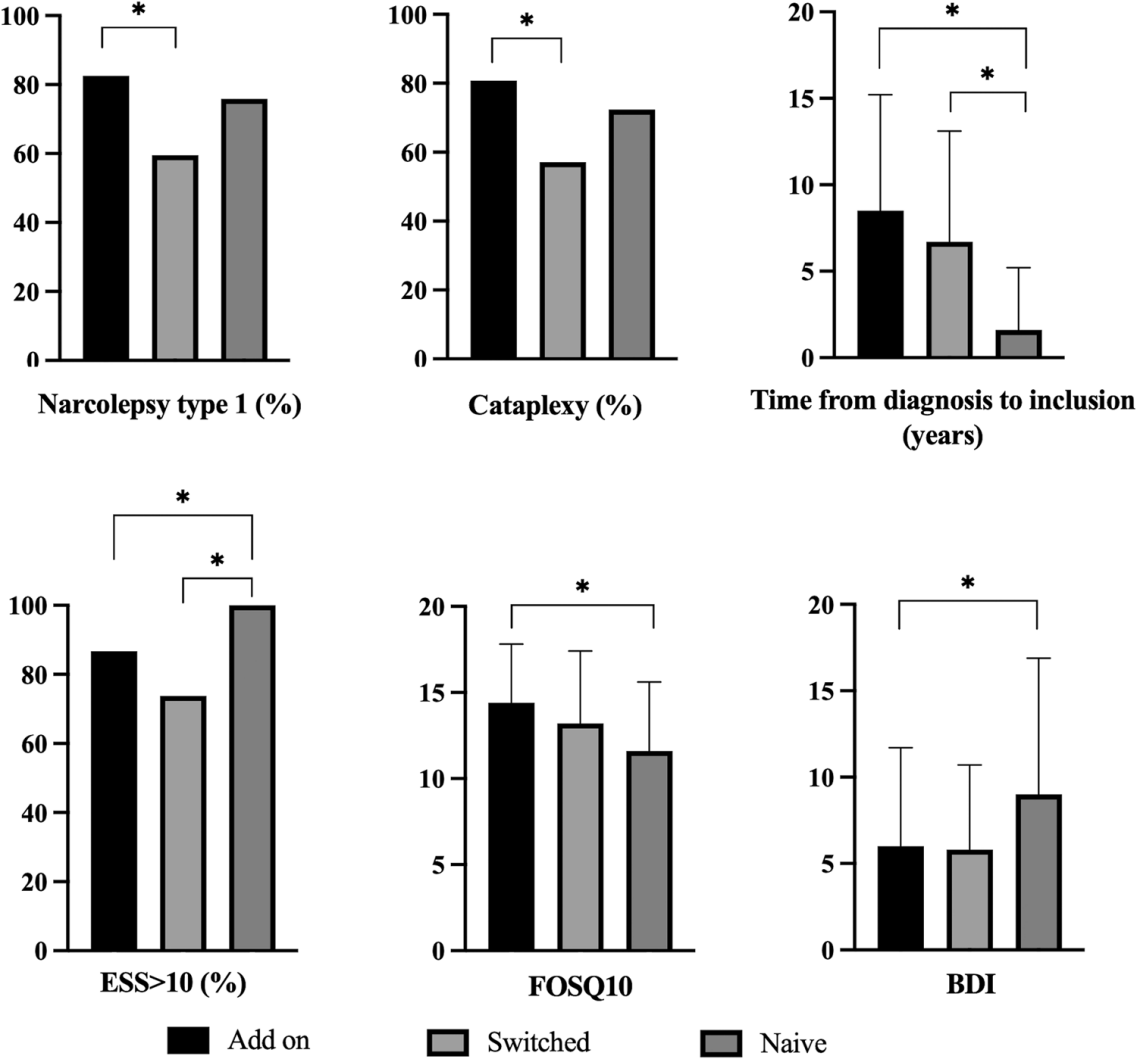
Significant post hoc comparisons between subgroups (“add-on group,” “switched group,” and “naive group”). Abbreviations: BDI, short version of the Beck Depression Inventory; ESS, Epworth Sleepiness Scale; FOSQ10, Functional Outcomes of Sleep Questionnaire

No other significant differences were observed in the comparison among the three subgroups. The most relevant significant results of post hoc comparisons are shown in Fig. 2. The results of the multivariate analysis substantially confirmed the findings of the univariate comparison. Further demographic and clinical details and comparison between groups are reported in Table 4.

**Table 4:** Comparisons between subgroups (“add-on group,” “switched group,” and “naïve group”)

	Add-on (n° 120, 62.8%)						Switched (n° 42, 22.0%)						Naive (n° 29, 15.2%)						Statistics		
	**n°**	**%**	**Mean**	**SD**	**Median**	**IQR**	**n°**	**%**	**Mean**	**SD**	**Median**	**IQR**	**n°**	**%**	**Mean**	**SD**	**Median**	**IQR**	**χ**^**2**^	***F***	***p***
Age (years)			43.1	16.3	42.9	27.3–56.1			41.7	16.2	42.9	30.0–50.2			41.0	15.3	41.7	28.1–53.8		0.260	0.772
BMI (kg/m^2^)			27.6	5.0	27.2	24.0–30.9			27.4	5.6	26.2	23.2–31.1			26.4	6.9	24.8	22.1–28.9		0.566	0.569
Sex (male, n°)	68	56.7					21	50.0					11	37.9					3.406		0.182
NT1 (n°)	99	82.5					25	59.5					22	75.9					9.126		0.010*
Age at diagnosis (years)			34.7	16.0	33.7	21.6–48.5			35.0	14.9	31.6	24.9–45.9			39.4	16.0	38.7	27.9–53.8		10.626	0.348
Time from diagnosis to inclusion (years)			8.5	6.7	7.1	3.4–12.9			6.7	6.4	6.2	1.1–10.9			1.6	3.6	0.1	0.0–0.3		142.424	< 0.001*
ESS (score)			14.8	4.1	15.0	12.0–18.0			15.1	5.4	16.0	10.0–20.0			16.8	3.6	17.0	14.0–19.0		24.745	0.087
ESS > 10 (n°)	104	86.7					31	73.8					29	100					9.866		0.007*
ESS > 15 (n°)	51	42.5					23	54.8					17	58.6					3.527		0.171
Cataplexy (n°)	97	80.8					24	57.1					21	72.4					9.222		0.010*
Sleep paralysis (n°)	75	62.5					18	42.9					22	75.9					8.507		0.014*
Sleep hallucinations (n°)	81	67.5					24	57.1					18	62.1					1.537		0.464
Disrupted nocturnal sleep (n°)	82	68.3					32	76.2					18	62.1					1.694		0.429
Automatic behavior (n°)	41	34.2					17	40.5					14	48.3					2.157		0.340
BDI (score)			6.0	5.7	6.5	2.0–10.0			5.8	4.9	5.0	2.0–8.0			9.0	7.9	6.5	3.5–12.0		32.814	0.040*
Mild depressive symptoms (BDI 5–7) (n°)	14	11.9					10	23.8					6	21.4					4.106		0.128
Moderate depressive symptoms (BDI 8–15) (n°)	34	28.8					11	26.2					7	24.1					0.237		0.888
Severe depressive symptoms (BDI 15–39) (n°)	8	6.8					2	4.8					6	21.4					6.900		0.032*
Comorbidities (n°)	76	63.3					29	69.0					16	55.2					1.423		0.491
CV comorbidity (n°)	37	30.8					9	21.4					9	31.0					1.426		0.490
Psychiatric comorbidity (n°)	27	22.5					12	28.6					7	24.1					0.627		0.731
Obesity (n°)	42	35					14	33.3					6	20.7					2.200		0.333
QoL “in health” (n°)	37	31.9					11	26.2					6	22.2					1.300		0.522
VAS QoL (score)			67.2	19.0	70	51.0–80.0			61.4	21.9	65.0	50.0–75.0			59.3	24.4	64.5	35.0–77.5		24.337	0.091
FOSQ10 (score)			14.4	3.4	15	11.5–17.2			13.2	4.2	14.5	9.8–16.5			11.6	4.0	11.8	7.8–14.3		72.770	0.001*

*BDI*, short version of the Beck Depression Inventory; *BMI*, body mass index; *CV*, cardiovascular; *EDS*, excessive daytime sleepiness; *ESS*, Epworth Sleepiness Scale; *EQ-5D-5L*, 5-level EQ-5D version; *FOSQ10*, Functional Outcomes of Sleep Questionnaire; *IQR*, interquartile range; NT1, narcolepsy type 1; *QoL*, quality of life; *SD*, standard deviation; *VAS*, visual analogue scale

^*^Parameters that reached statistical significance

## Discussion

Herein, we provide a detailed picture of a large population of Italian narcoleptic patients candidate for treatment with pitolisant followed in 14 Italian sleep centers that share similar diagnostic and therapeutic approaches in the management of narcolepsy.

Our study showed as the first finding that pitolisant was more commonly prescribed in patients affected by NT1 (76.4% of the total sample), likely due to its beneficial effect on both EDS and cataplexy, and possibly reflecting the lower prevalence of NT2 compared to NT1 in the context of central disorders of hypersomnolence [27]. The low representation of NT2 may be related to the characteristics of third-level sleep centers participating to the study, who generally have more severe patients’ referrals [28]. Regarding clinical data of our cohort, it is of interest that dyssomnia was identified in most cases (69.1%), confirming that disrupted nocturnal sleep is an intrinsic feature of narcolepsy that does not impact on the sleep specialist’s therapeutical choice. We observed a mean age at diagnosis of 35.5 years consistent with the bimodal distribution of narcolepsy onset, with a main peak in the teens [29].

We found that most patients complained of severe impairment of daytime functioning, with major impact on their ability to carry out daily activities and on psychological status. From this point of view, our study confirms that narcolepsy severely impacts the quality of life and wellbeing of patients [30, 31]. In particular, in our cohort, the burden of sleepiness-related impairment on the ability to conduct daily and recreational activities, measured by means of FOSQ10, was higher in drug naïve patients. Similarly, the percentage of patients complaining of severe depressive symptoms was significantly higher in the treatment-naive group (21.4%) compared to treated patients (respectively 4.8% in the switched and 6.8% in the add-on groups). This data may indicate the effectiveness of anti-narcoleptic treatment on the psychological consequences of the disease or may suggest a direct antidepressant effect of anti-cataplectic medications. Yet, the higher prevalence of depressive symptoms in drug-naïve patients may suggest also that sleep specialists are more likely to prescribe pitolisant as first line to people with comorbid depression instead of other currently prescribed medications acting on the dopaminergic or gabaergic neurotransmission. As a matter of fact, our study underlines that psychiatric disorders are frequent comorbidities and may represent a diagnostic and therapeutic challenge [32]. Thus, a prompt diagnosis and treatment of narcolepsy can counteract the impact of the disease on patients’ quality of life. From this point of view, diagnostic delay is a well-known issue in the management of narcoleptic patients [5, 29]. “Red Flags” for narcolepsy were recently proposed by a multidisciplinary panel of experts, supported by the “Associazione Italiana Narcolettici e Ipersonni” (AIN, www.narcolessia.org) to help physicians heighten their awareness on typical clues [33].

Another evidence of our study is the high prevalence of cardiovascular comorbidities in narcolepsy. We found that 28.8% of our sample, regardless of therapy, presented a cardiovascular disease. This prevalence is higher compared to the general population of the same age. Moreover, a high percentage of patients suffered from sleep apnea, hypercholesterolemia, diabetes, and obesity, which concur to worsen cardiovascular risk. These findings are in line with current knowledge on narcolepsy [3], but may also reflect the preference, in clinical practice, to prescribe pitolisant in patients with high cardiovascular burden, given its known negligible impact on the cardiovascular system [34]. Therefore, the high prevalence of cardiovascular comorbidities may be over-estimated in our sample.

The second aim of our study was to compare patients according to their medication scheme at pitolisant initiation, following the approach of the sleep specialists managing patients with narcolepsy. The naïve-group (drug-naïve at the time of data analysis) are newly diagnosed patients or patients likely responsive to pitolisant as first-line treatment; the patients of the switched group are more complex patients, poorly responsive to a previous treatment but in whom the monotherapy still appears reasonable; finally, the add-on group includes patients with the highest level of complexity of the pharmacological management, in which monotherapy failed to reach an adequate control of narcoleptic symptoms. First, we observed that pitolisant is more commonly prescribed as an add-on treatment, with 29.8% of our cohort already under two anti-narcoleptic medications and up to 15.7% taking three or more anti-narcoleptic drugs. The polytherapy applied in most cases pinpoints the clinical need to adopt a complex pharmacological management to reach adequate symptoms’ control. Patients of the add-on group were more commonly affected by NT1, showed a longer disease duration, and had a higher (although not significantly) prevalence of comorbidities suggesting that high disease severity might be a factor imposing the need of a complex drug management. At the same time, given the novelty of pitolisant at the time of study enrollment, clinicians may have preferred its use in complex cases that were still symptomatic despite the previous use of different drug combinations, as highlighted by the high prevalence (86.7%) of patients complaining EDS in the “add-on group”. On the other hand, it should be taken into account that, given the novelty of pitolisant, physicians could have preferred prescribing it as an add-on rather than as first-line therapy in patients with narcolepsy. However, the polytherapy approach may take advantage of different drug specificities (e.g., wake-promoting, or sleep-stabilizing) to reach an adjunctive or synergistic effect [35], or could enable effectiveness with a lower dose of different medications avoiding adverse events.

Among wake-promoting drugs, pitolisant is the first antagonist/inverse agonist of the histamine H3 autoreceptor (H3-R) proposed for clinical use. Blocking the H3-R, pitolisant enhances histaminergic release in a dose-dependent manner and, in animal models, it indirectly promotes the increase of dopamine and acetylcholine release, but not in the nucleus accumbens [36]. Modafinil has several effects on catecholamine systems in the brain, resulting in increased levels of dopamine, norepinephrine, serotonin, and histamine; activation of the orexinergic system; and decreased GABA[37]. Solriamfetol had been hypothesized to act mainly through a selective dopamine and norepinephrine reuptake inhibition [38]. Sodium oxybate is a sleep-stabilizing drug. It is the sodium salt of gamma-hydroxybutyrate (GBH), an endogenous metabolite of the neurotransmitter GABA; the therapeutic effects of sodium oxybate are mediated to GABA-B receptor agonist activity, resulting in enhancement of the slow wave sleep, an increase in sleep efficiency, and prolonged REM latency [39].

So far, few studies investigated the efficacy and safety of combined therapy in narcolepsy, but recent European guidelines promote the use of polytherapy as a second-line option [21]. In the light of the pitolisant prescribing scheme, we observed different levels of complexity of the pharmacological management of patients affected by narcolepsy. In the near future, a larger choice of medications will be available to manage EDS, cataplexy, and the vast spectrum of symptoms associated with narcolepsy. From this point of view, rational polytherapy will play a pivotal role in the management of patients affected by narcolepsy, allowing a more personalized treatment.

Our study has some limitations. First, our data cannot be generalized to the entire Italian narcolepsy population. In fact, patients enrolled in the current study were all candidate for pitolisant treatment, thus most probably favoring the inclusion of cases of difficult management who did not have adequate and stable symptoms’ control. Only in a minority of cases, pitolisant was prescribed as first-line treatment, most notably in NT1 patients in order to try to control EDS and cataplexy. Second, most of the data were collected by means of validated self-administered questionnaires, which provide only subjective measures of sleep and sleepiness, as required by the PASS pitolisant protocol. However, these tools are extensively adopted in clinical research and have all been rigorously validated. Moreover, data were collected from all the major national sleep centers with strong expertise in the field. Another limitation of the current study is the cross-sectional design, which does not permit to report the outcome of patients being prescribed with pitolisant; thus, further studies will clarify, and possibly support, the appropriateness of prescription choice, as well as the efficacy and safety of pitolisant in the context of polytherapy in a real-world setting.

Despite these drawbacks, our study provides extensive clinical data on a large cohort of Italian narcoleptic patients candidate for pitolisant treatment in a real-world setting. This study also reinforced the Italian network of sleep centers involved in the management of this rare disease, and the shared methodologies applied for the diagnostic work-up and follow-up of patients do constitute a step forward for the establishment of an Italian national database for narcolepsy.

## Data Availability

The data that support the findings of this study are available from Bioprojet. Restrictions apply to the availability of these data, which were used under license for this study.
